# Senkyunolide I and Danshensu in combination ameliorate post-MI myocardial fibrosis by regulating *TGF-β1/PAI-1* in mice and heart organoids

**DOI:** 10.3389/fphar.2026.1862471

**Published:** 2026-07-08

**Authors:** Siwen Fan, Mengxu Shi, Yuhan Zhao, Wenan Nie, Yixin Yao, Linhong Han, Shuang He, Guangxu Xiao, Yan Zhu

**Affiliations:** 1 State Key Laboratory of Chinese Medicine Modernization, Tianjin University of Traditional Chinese Medicine, Tianjin, China; 2 State Key Laboratory of Component-Based Chinese Medicine, Tianjin University of Traditional Chinese Medicine, Tianjin, China; 3 Haihe Laboratory of Modern Chinese Medicine, Tianjin, China; 4 Department of Pharmacy, China Academy of Chinese Medical Sciences, Guang’anmen Hospital Jinan Hospital, Jinan, China; 5 College of Pharmaceutical Engineering of Traditional Chinese Medicine, Tianjin University of Traditional Chinese Medicine, Tianjin, China; 6 First Teaching Hospital of Tianjin University of Traditional Chinese Medicine, Tianjin, China; 7 National Clinical Research Center for Chinese Medicine, Tianjin, China

**Keywords:** heart organoid, myocardial fibrosis, myocardial infarction, PAI-1, Senkyunolide I, Danshensu, Guanxinning injection

## Abstract

**Background:**

Myocardial fibrosis following myocardial infarction (MI) is a critical determinant of long-term clinical outcomes in heart disease. However, this central pathological process in the progression from MI to heart failure is not fully understood, posing challenges for targeted therapeutic options.

**Purpose:**

This study aimed to identify new targets critical to myocardial fibrosis after MI and to evaluate the potential of Guanxinning injection (GXNI, a combination of Danshen and Chuanxiong) to suppress post-MI myocardial fibrosis.

**Methods:**

A MI mouse model was established by permanent ligation of the left anterior descending artery. Therapeutic effects were assessed by echocardiography and histopathology. HPLC chromatography, Network pharmacology, molecular docking, two-dimensional primary cell cultures, and iPS-induced cardiac organoid models were employed to identify active components and investigate underlying mechanisms.

**Results:**

GXNI reduced myocardial fibrosis and injury, improving cardiac function. Network pharmacology and molecular docking identified Senkyunolide I (SENI) and Danshensu (DSS) as the key active components acting via the TGF-β1/PAI-1 pathway. Thermal shift assay demonstrated direct binding of SENI and DSS to PAI-1. *In vitro* and cryoinjury cardiac organoid models confirmed that GXNI and the SENI-DSS combination inhibited fibroblast activation and TGF-β1/PAI-1 expression, reducing fibrosis.

**Conclusion:**

We demonstrate for the first time that the TGF-β1/PAI-1 signaling pathway plays a critical role in post-MI myocardial fibrosis, and GXNI attenuates MI via this pathway. Secondly, the active components of GXNI, SENI and DSS, act combinatorially to enhance the anti-fibrotic therapeutic efficacy. Furthermore, cryoinjury cardiac organoids serve as a powerful platform for investigating the mechanisms underlying myocardial infarction.

## Graphical Abstract

**Figure:**
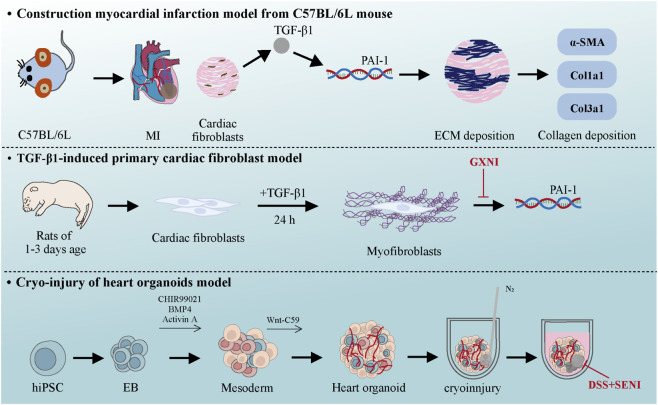

## Highlights

- Cryoinjury to hiPSC-derived heart organoid mimics an in vivo-like myocardial infarction
- The TGF-β1/PAI-1 axis critically safeguards against myocardial infarction by modulating myocardial fibrosis
- DSS and SENI combination targets PAI-1 to alleviate myocardial fibrosis.

## 1 Introduction

In middle-income countries, cardiovascular disease prevalence is double that in high-income countries, owing to environmental pollution, unhealthy lifestyles, obesity, and hypertension, resulting in high mortality and a significant financial burden from mismatched treatment needs and resources (Arnett et al., 2019; Han et al., 2022; Timmis et al., 2022). Among these, myocardial infarction (MI) is the most lethal of cardiovascular diseases (Prabhu and Frangogiannis, 2016; Virani et al., 2021). Despite current improvements in treatment, patients with myocardial infarction still face a high risk of death, and treating and improving myocardial infarction remains a challenge (Chapman et al., 2020).

Myocardial infarction is mainly due to necrosis of myocardial cells due to persistent ischemia and hypoxia as a result of arterial plaque rupture or embolism (Frangogiannis, 2015). Myocardial infarction leads to abnormal proliferation of cardiac fibroblasts (CFs) and excessive deposition of ECM. Cardiac fibroblasts are activated into myofibroblasts, and the hallmark of their activation is the synthesis and expression of α-SMA. Activated myofibroblasts migrate to the infarcted area and can simultaneously promote collagen I and III production and deposition in the area of myocardial infarction (Travers et al., 2016). Although fibroblasts are involved in the repair process early on, their persistence and excessive activation can disrupt cardiomyocytes’ normal contraction and conduction (Aghajanian et al., 2019). At the same time, the hardening of the ventricular walls in fibrotic heart tissue and reduced compliance can lead to decreased cardiac function and eventual heart failure, leading to death (Shinde and Frangogiannis, 2014; Venugopal et al., 2022). A recent relevant study that used injectable biomimetic microtissues for MI repair also addresses ECM remodeling and fibrosis reduction (Yao et al., 2026). It is crucial to explore the importance of improving myocardial fibrosis after myocardial infarction to reduce ventricular remodeling and prevent heart failure.

Guanxinning injection (GXNI) composed of Chinese Materia Medica standards extracted from Radix Salvia miltiorrhiza and Rhizoma Ligusticum chuanxiong, is a traditional Chinese medicine prescription. Traditionally, GXNI has been applied to promote blood circulation, remove blood stasis, promote pulse, and nourish the heart. GXNI is approved by the China Food and Drug Administration (CFDA) and is widely applied in clinical practice to treat myocardial infarction, coronary heart disease, and angina pectoris (Jia et al., 2013; Wang A. et al., 2022; Wang C. L. et al., 2022; Xiao et al., 2022). In a randomized, multicenter, and placebo-controlled clinical trial study with 42 coronary heart disease complicated with congestive heart patients, GXNI significantly improved of cardiac function and hemorheology index (Wang and Gu, 2026). Clinical efficacy evaluation showed that co-treatment with GXNI and conventional Western medicine can improve the therapeutic outcomes of IS by reducing myocardial remodeling and oxidative stress (Hu et al., 2025). Pharmacological evidence further indicates that GXNI improves myocardial injury caused by heart failure by regulating p38/c-Fos/Mmp1 in an *in vitro* 3D heart-like organ model and an *in vitro* aortic arch constriction (TAC) mouse model, and showed that GXNI is effective in treating myocardial fibrosis (Fan et al., 2023). GXNI comprises two Chinese herbs, including *Salvia miltiorrhiza* Bge. (Dan-Shen) and *Ligusticum chuanxiong* Hort. (Chuan-Xiong) in a ratio of 1:1. Furthermore, in previous studies, GXNI in each plant was shown to be effective for myocardial infarction. For example, Dan-Shen can improve myocardial ischemia-reperfusion injury through downregulating the CXCR1-NF-κB-COX-2/ICAM-1/VCAM-1 pathway, inhibiting inflammatory factor release and leukocyte infiltration (Xiao et al., 2022). A bioactive alkaloid derived from Chuan-Xiong alleviates ventricular remodeling after myocardial infarction by regulating Sirt1/p300/Yy1/sST2 signaling axis (Liu et al., 2026). Although the above findings provided the initial evidence of GXNI for treating myocardial fibrosis after myocardial infarction, its specific therapeutic efficacy and mechanism towards different heart diseases remain unclear.

It is noteworthy that traditional animal and two-dimensional cell models are insufficient to replicate human myocardial infarction fully. Therefore, a cryoinjury human iPS-induced cardiac organoid model was used. In this injury model, placing a liquid nitrogen-cooled probe on the cardiac organoid causes cell death, activates the inflammatory response, and deposits scar tissue containing fibrous collagen, similar to myocardial infarction in the human heart (Constanty et al., 2025). Therefore, we used the mouse myocardial infarction model, TGF-β1-induced two-dimensional cardiac fibrosis model, and cryoinjured cardiac organoid model to simultaneously evaluate the therapeutic effects of GXNI on post-myocardial infarction cardiac fibrosis. Using an integrated strategy of network pharmacology and molecular docking analysis, we elucidated the drug’s mechanism of action and key active components. Our study showed that GXNI ameliorates myocardial fibrosis after myocardial infarction mainly by modulating TGF-β1/PAI-1, and the main active components responsible for its action are Danshensu and Senkyunolide I.

## 2 Materials and methods

### 2.1 Drugs and reagents

Guanxinning injection (CFDA drug approval number Z13020779) was provided by China Shineway Pharmaceutical Group Ltd. (Shijiazhuang, China, batch number 171122B2). hiPSC medium (PGM1, CA1014500), hiPSC cell culture coating solution (CA3003100), and hiPSC cell digestion solution (CA1023100) were purchased from Beijing Cellapy Biotechnology Co., LTD (Beijing, China). Bicinchoninic acid protein concentration assay kit (BCA, PC0020), High-efficiency RIPA tissue/cell lysate (R0010), Tris Buffered saline Tween (TBS-T, T1081), Tris-Glycine Running Buffer (T1070), and Western blot Transfer Buffer (D1060) were purchased from Solarbio (Beijing, China). Pierce™ ECL Western Blotting Substrate (32,109) was purchased from Thermo (New York, United States). SDS-PAGE Protein Loading Buffer was purchased from Beyotime (P0015L, Shanghai, China). Anti-vimentin antibody (ab24525) was purchased from Abcam (Beijing, China). Anti-TGF-β1 antibody (21898-1-AP) was purchased from Proteintech (Wuhan, China). Anti-α-SMA antibody (ab7817) and anti-PAI-1 antibody (ab182973) were purchased from Abcam (Beijing, China). Anti-GAPDH antibody (14C10) was purchased from Cell Signaling Technology (Danvers, United States). HRP Goat anti-Rabbit (RS0002) was purchased from Immunoway (Plano, United States). The hematoxylin and eosin (H&E) staining kit was purchased from Beyotime Biotechnology (C0105, Shanghai, China). Transcriptor First Strand cDNA Synthesis Kit was purchased from Roche (04897030001, Mannheim, Germany). Bestar™ qPCR MasterMix was purchased from DBI Bioscience (DBI-2043, Shanghai, China).

### 2.2 Experimental animals

All animal experiment protocols were designed in accordance with the principles of the Basel Declaration and recommendations of the Care and Use of Laboratory Animals promulgated by the Ministry of Science and Technology of China. They were implemented after approval by the Laboratory Animal Ethics Committee of Tianjin University of Traditional Chinese Medicine (License number: TCM-LAEC2021216). Wild-type male C57BL/6J mice aged 8–10 weeks were purchased from Beijing Vital River Laboratory Animal Technology Co., Ltd. (Beijing, China, Certificate no.: SCXK Jing 2020-0004). Mice were reared in cages where they could eat and drink freely under conditions of 22 °C ± 2 °C and relative humidity of 40% ± 5%, and maintained a 12 h-light/dark cycle. Myocardial infarction (MI) was induced in mice following a previously described protocol (Xiao et al., 2019). In brief, after anesthesia, each mouse was intubated and connected to a ventilator. A thoracotomy was then performed to expose the heart, and the left anterior descending (LAD) coronary artery adjacent to the left atrial appendage (LAA) was ligated using a 7-0 monofilament suture. The surgical wound was subsequently disinfected and sutured. The mice were randomly allocated into six groups: sham, I/R (saline), GXNI at 1 mL/kg, GXNI at 3 mL/kg, GXNI at 9 mL/kg, and the positive control drug metoprolol (1 mL/kg). In the sham group, animals underwent thoracotomy, and the suture thread was passed through the ligation site without actual ligation. All treatments or normal saline were administered via the tail vein.

### 2.3 Echocardiography and hemodynamic examination

Echocardiography and hemodynamic examination were performed as we described previously (Fan et al., 2023). Briefly, left ventricular function and coronary blood flow were measured using a small-animal ultrasound imaging system (VEVO 2100, VisualSonics, Toronto, ON, Canada) at 1, 14, and 28 days after LAD surgery. The fully anesthetized mice were fixed on a heated imaging platform, and the appropriate amount of chelating agent was applied to the limbs and chest for echocardiographic and electrocardiographic detection. The ultrasound system was set to M-mode to measure cardiac activity. The short-axis shortening (FS) and the ejection fraction (EF) of the heart were recorded in each group. Then, coronary blood flow was determined using the Color Doppler mode, which was evaluated by aortic valve (AV) peak velocity, AV peak pressure, and aorta velocity-time integral mean velocity (AoV VTI).

### 2.4 Immunohistochemistry

After paraffin removal, heart tissue sections were rinsed 3 times in PBS-T solution and incubated with 3% peroxide methanol for 10 min to block the endogenous peroxidase. The sections were then blocked with 10% bovine serum albumin solution at 37 °C for 1 h, incubated with primary antibody (anti-α-SMA, 1:400; anti-TGF-β1, 1:400; and anti-PAI-1, 1:200 diluted in blocking solution) for 2 h, washed 3 times with PBS-T solution for 5 min, and incubated with the corresponding secondary antibody at 37 °C for 1 h. The immunoreactivity of α-SMA, TGF-β1, and PAI-1 proteins was visualized by 3,3′-diaminobenzidine tetrahydrochloride hydrate and stained with hematoxylin and fixed. Finally, the expression of α-SMA, TGF-β1, and PAI-1 was quantified by an automated quantitative pathology imaging system (Vectra 3, PerkinElmer Inc.).

### 2.5 Primary cardiac fibroblast cell culture

The method for primary cardiac fibroblast cell isolation was described previously (Ackers-Johnson et al., 2016). Immediately after sacrificing the neonatal SD rats, the hearts were removed, washed in ice-cold PBS, cut into small pieces, and digested at 37 °C in PBS containing 10% fetal bovine serum, 0.1% type I collagenase, and 0.06% trypsin for 5 min, for a total of 6 times. Cell suspensions were centrifuged at 1,000 r/min for 10 min and resuspended in DEME medium containing 10% fetal bovine serum, filtered to remove cell clumps, and evenly plated in culture flasks for 90 min. Cardiomyocytes were separated using the differential attachment method, retaining the adherent fibroblasts. Fibroblast identification was performed using Vimentin immunofluorescence staining.

### 2.6 Establishment of cardiac fibrosis model and drug administration

As previously described (Fan et al., 2025a), cardiac fibroblasts were cultured in DMEM medium containing TGF-β1 for 24 h to establish cardiac fibrosis. To investigate the GXNI or DSS-SENI effects, cardiac fibroblasts were divided into groups including control, TGF-β1 (10 ng/mL or 20 ng/mL), GXNI (1 μL/mL, 3 μL/mL, and 9 μL/mL for 24 h with TGF-β1), 2.6 mg/mL DSS and 0.4 mg/mL SENI mixed solution (1 μL/mL, 3 μL/mL, and 9 μL/mL for 24 h with TGF-β1), MP (1 μL/mL MP for 24 h with TGF-β1), and Tiplaxtinin (10 μM Tiplaxtinin for 24 h with TGF-β1). All the groups were treated after 1 h of drug pretreatment, except for the control group, which was treated with normal medium.

### 2.7 iPSC stem cell culture and differentiation into cardiac organoids

Human-induced pluripotent stem cells (hiPSCs) line B1 (CA4025106, Beijing, China) were used in this study. hiPSCs were cultured in 6-well plates coated with cell seeding solution. Using PGM1 medium, the cells were cultured in an incubator at 37 °C with 5% CO_2_ until they reached 60%–80% confluency. Then, they were digested with Stem Cell Digestive Solution and passaged into new 6-well plates. Heart organoid differentiation was described previously (Lewis-Israeli et al., 2021). Briefly, after dissociation, hiPSCs were resuspended in PGM1 medium containing 2 μM ROCK inhibitor Thiazovivin (Merck Millipore) on day −2. About 10,000 cells were seeded in a 96-well round-bottom plate (MS-9096UZ, Sumitomo Bakelite Co., Ltd., Tokyo, Japan). The plate was then centrifuged at 100 *g* for 3 min and placed in an incubator at 37 °C, 5% CO_2_. On day −1, cells were equilibrated in Essential 8 Flex medium. From day 0, a directed differentiation was initiated using CHIR99021, BMP4, and Activin A in RPMI/B-27 (-insulin). Wnt-C59 was added on day 2, followed by media changes every 48 h. Insulin was reintroduced on day 6. A second pulse of CHIR99021 was applied on day 7, and the organoids were maintained until analysis.

### 2.8 Cryo-injury of heart organoids and drug administration

As previously described (Hofbauer et al., 2021), cardiac organoids were placed in a culture dish devoid of medium before cryo-injury. A brief contact was made with a liquid-nitrogen-chilled steel rod, after which the organoids were returned to the maintenance medium for further culture and analysis. To investigate the DSS-SENI effects, cardiac organoids were divided into 8 groups: control, N_2_ model, DSS-SENI (3 μL/mL), Tiplaxtinin (10 μM), TGF-β1 (10 ng/mL) + DSS-SENI (3 μL/mL), 3 μL/mL GXNI, 9 μL/mL GXNI, and Metoprolol (MP, 1 μL/mL). After 6 h of drug pretreatment, all the groups were cryo-injured with N_2_ for 72 h, except for the control group, which was treated with normal medium.

### 2.9 Immunofluorescence (IF)

Primary cardiac fibroblasts on a 96-well plate and cardiac organoids were fixed with 4% paraformaldehyde solution and blocked with 5% FBS for 2 h, incubated with primary antibody (anti-α-SMA, 1:400; anti-vimentin, 1:100; anti-TGF-β1, 1:400; and anti-PAI-1,1:200) overnight at 4 °C, washed with PBS-T, and incubated with the corresponding fluorescent secondary antibody (Alexa Fluor 555,1:1,000; Alexa Fluor 488, 1:1,000; or Alexa Fluor 647, 1:1,000) and Hoechst 33,342 for 2 h at room temperature. Finally, after washing the cells with PBS-T in the dark, the Operetta high-content analysis system (PerkinElmer Inc., United States) was used to quantify the number of cardiac fibroblasts and their fluorescence intensity.

### 2.10 Real-time reverse transcription polymerase chain reaction assay

Total RNA was extracted from heart tissue using TRIzol™ Reagent. The cDNA was obtained by reverse transcription using Transcriptor First Strand cDNA Synthesis Kit, added to the PCR strip tubes with Bestar™ qPCR MasterMix and primers, and amplified in a real-time PCR system (LightCycler®480, Roche, Germany) to detect its mRNA expression level. The genes to be detected in this experiment include *Col1a1, Col3a1, α-SMA,* and *TGF-β1*, using glyceraldehyde 3-phosphate dehydrogenase (*GAPDH*) as a standard. Mouse and human gene primers for the above genes (Tables 1, 2) were synthesized by Sangon Company (Shanghai, China).

**TABLE 1:** Primer sequences of mouse genes.

Primer name	Primer direction	Primer sequence (5′–3′)
*Col1a1*	Sense	TGA​ACG​TGG​TGT​ACA​AGG​TC
*Col1a1*	Antisense	CCA​TCT​TTA​CCA​GGA​GAA​CCA​T
*Col3a1*	Sense	GAA​AGA​ATG​GGG​AGA​CTG​GAC
*Col3a1*	Antisense	TAC​CAG​GTA​TGC​CTT​GTA​ATC​C
*α-SMA*	Sense	CGT​GGC​TAT​TCC​TTC​GTG​ACT​ACT​G
*α-SMA*	Antisense	CGT​CAG​GCA​GTT​CGT​AGC​TCT​TC
*Tgf-β1*	Sense	CCA​GAT​CCT​GTC​CAA​ACT​AAG​G
*Tgf-β1*	Antisense	CTC​TTT​AGC​ATA​GTA​GTC​CGC​T
*Gapdh*	Sense	TGG​TGA​AGC​AGG​CAT​CTG​AG
*Gapdh*	Antisense	TGC​TGT​TGA​AGT​CGC​AGG​AG

**TABLE 2:** Primer sequences of human genes.

Primer name	Primer direction	Primer sequence (5′–3′)
*COL1A1*	Sense	GTG​CGA​TGA​CGT​GAT​CTG​TGA
*COL1A1*	Antisense	CGG​TGG​TTT​CTT​GGT​CGG​T
*COL3A1*	Sense	GGA​GCT​GGC​TAC​TTC​TCG​C
*COL3A1*	Antisense	GGG​AAC​ATC​CTC​CTT​CAA​CAG
*α-SMA*	Sense	GAG​AGG​AGC​AAA​ATC​TGT​CCG
*α-SMA*	Antisense	GGG​GGA​ATT​ATC​TTT​CCT​GGT​CC
*TGF-β1*	Sense	GCA​ACA​ATT​CCT​GGC​GAT​ACC​TC
*TGF-β1*	Antisense	CCT​CCA​CGG​CTC​AAC​CAC​TG
*PAI-1*	Sense	GGC​TGG​TGC​TGG​TGA​ATG​C
*PAI-1*	Antisense	AGT​GCT​GCC​GTC​TGA​TTT​GTG
*GAPDH*	Sense	CTG​GGC​TAC​ACT​GAG​CAC​C
*GAPDH*	Antisense	AAG​TGG​TCG​TTG​AGG​GCA​ATG

### 2.11 Western blotting

As we previously described (Xiao et al., 2022), the heart tissue was lysed using a high-efficiency RIPA tissue/cell lysate, and protein concentration in the supernatant was determined by the BCA kit. The protein samples were separated with SDS-polyacrylamide gel electrophoresis and transferred to the PVDF membrane, blocked, and incubated with the primary antibody overnight at 4 °C, washed with TBS-T, and incubated with the corresponding secondary antibody at room temperature for 2 h. Finally, the target protein was captured in the gel imaging system after the development of the ECL Western blotting substrate. The protein expression levels of PAI-1 were analyzed by ImageJ and standardized to GAPDH.

### 2.12 Histology

Cardiac organoid sections and mouse myocardial tissue sections were dewaxed and then placed in an automatic staining machine (ClearVue, Thermo Fisher Scientific, Waltham, MA, United States) for H&E staining. The sections were soaked in tissue deparaffinization solution 3 times, each for 5 min, followed by anhydrous alcohol twice, each for 5 min. Then, they were soaked in 95%, 85%, and 75% alcohol for 1 min each and differentiated in 1% hydrochloric acid alcohol for 5 s. After staining in hematoxylin solution for 7 min, they were dyed with an aqueous eosin solution for 3 min, then sequentially soaked in 75%, 85%, 95%, and 100% alcohol and xylene for 1 min each, after washing with running water for 1 min. Finally, the slides were sealed with neutral resin glue. Additionally, after deparaffinization of the sections, collagen deposition in the tissue was captured using a Masson trichromatic staining kit (G1346, Solarbio, Beijing, China) following the manufacturer’s instructions to assess its fibrosis.

For immunofluorescence staining, the mouse heart tissue was fixed in 4% paraformaldehyde for at least 24 h, dehydrated with an automatic dehydrator (Excelsior, Thermo Fisher Scientific, Waltham, MA, United States), and embedded in paraffin. The embedded tissues were cut into 5 µm-thick slices using a manual microtome (HM355S, Thermo Fisher Scientific, Waltham, MA, United States). After deparaffinization, the sections were incubated in 3% H_2_O_2_ for 10 min to block endogenous peroxidase. Next, the tissue sections were blocked in 10% BAS blocking solution for 1 h and incubated in the primary antibody solution (α-SMA, 1:400; TGF-β1, 1:400; PAI-1, 1:200) diluted with blocking solution for 2 h at 37 °C. After washing 3 times with PBS, the sections were dropwise added with biotinylated goat anti-rabbit IgG (1:800) and incubated at 37 °C for 40 min. Finally, after color development using the DAB kit, they were stained with hematoxylin and sealed with neutral resin glue.

The internal tissue structure of the cardiac microspheres, the pathological changes in mouse myocardial tissue, and the protein expression levels of α-SMA, TGF-β1, and PAI-1 in tissue sections were photographed and quantified using an automated quantitative pathology imaging system (Vectra 3, PerkinElmer).

### 2.13 Statistical analysis

Data were expressed as mean ± SD. GraphPad Prism 7 software (GraphPad Software, Inc., La Jolla, CA, United States) was used for statistical analysis. The student’s two-tailed t-test was utilized to compare two groups, while multiple groups comparison was performed with one-way analysis of variance (ANOVA). A value of P < 0.05 was defined as statistically significant.

## 3 Results

### 3.1 GXNI ameliorated the cardiac dysfunction in MI mice

**FIGURE 1:**
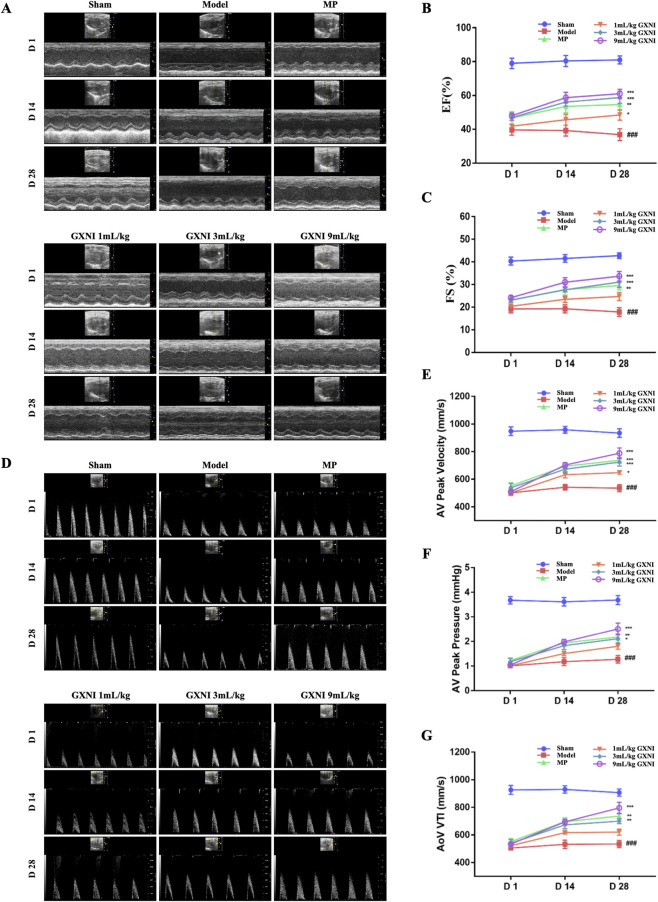
The effect of GXNI on echocardiographic characteristics of cardiac function and coronary blood flow in MI mice. Cardiac function and coronary blood flow were assessed in each group of mice on days 1, 14, and 28 after permanent ligation of the LAD artery. **(A)** Representative echocardiographic images of the sham, model, GXNI 1 mL/kg, GXNI 3 mL/kg, GXNI 9 mL/kg, and MP (1 mL/kg)-treated groups. **(B)** Quantitation of EF%. **(C)** Quantitation of FS %. **(D)** Representative echocardiography images of the coronary blood flow determined in color Doppler mode of each group. **(E)** Quantification of AV peak velocity. **(F)** Quantification of AV peak pressure. **(G)** Quantification of AoV VTI. Values were expressed as mean ± SD (n = 5). ^###^ *P* < 0.001 vs. sham, *P < 0.05, **P < 0.01, ***P < 0.001 vs. model (EF, ejection fraction; FS, fractional shortening; AV, aortic valve; AoV VTI, aorta velocity–time integral mean velocity).

On days 1, 14, and 28 after permanent ligation of the LAD artery in mice, cardiac function was evaluated by echocardiography (Figure 1A). Compared with the Sham, the ventricular chambers of the model mice were significantly enlarged. The ejection fraction (EF) and fractional shortening (FS) of the heart were significantly reduced in the model mice, indicating severe cardiac dysfunction (Figures 1B,C). GXNI at 1, 3, and 9 mL/kg and MP (1 mg/kg) all markedly improved ventricular chamber dilation and significantly increased EF in the model mice (Figure 1B). The FS in the 3 and 9 mL/kg GXNI and MP-treated mice were also significantly improved compared with the model mice, but 1 mL/kg GXNI had no significant effect. Color Doppler of coronary blood flow results showed that the AV peak velocity, AV peak pressure, and AoV VTI of the model mice were remarkably lower than those in the sham mice (Figures 1D–G). However, the AV peak velocity decrease by MI was effectively improved by GXNI (1, 3, and 9 mL/kg) and MP (Figure 1E). The 3 and 9 mL/kg GXNI and MP also significantly increased AV peak pressure and AOV VTI in the model mice, but the effect of 1 mL/kg GXNI was insignificant (Figures 1F,G). In conclusion, these results suggest that GXNI significantly alleviates cardiac dysfunction caused by MI and exerts cardioprotective effects.

### 3.2 GXNI attenuates myocardial injury and reduces fibrosis in MI mice

**FIGURE 2:**
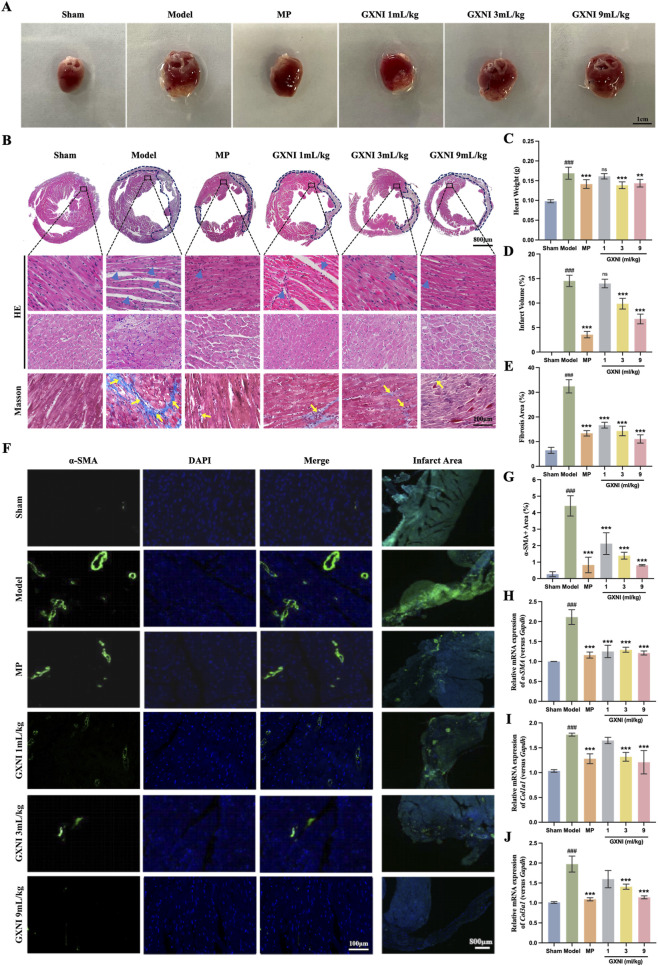
GXNI reduced heart weight, infarct area, and collagen deposition of MI mice. The heart tissues were removed after 28 days for pathological and statistical analyses, including sham, MI model, MP, GXNI 1 mL/kg, GXNI 3 mL/kg, and GXNI 9 mL/kg-treated mice. **(A)** Representative images of heart samples from each group. **(B)** Representative images of H&E staining and Masson staining results, where myocardial damage is indicated by yellow arrows and collagen deposition is indicated by blue arrows. The upper panel is panoramic scan images of myocardial tissue in cross-section. Normal myocardial tissue was red, while the infarcted area was white. **(C)** Quantification of heart weight in each group. **(D)** Quantification of infarct volume in each group. **(E)** Quantification of the area of collagen deposition in each group (n = 5). **(F)** Representative images of α-SMA immunofluorescence staining in non-infarcted areas and infarcted areas. **(G)** Quantification of α-SMA positivity in mouse heart tissue. **(H)** Gene expression of *α-SMA* in mouse heart tissue. **(I)** Gene expression of *Col1a1* in mouse heart tissue. **(J)** Gene expression of *Col3a1* in mouse heart tissue. Data were expressed as mean ± SD. ^###^ *P* < 0.001 vs. sham group, ^*^ *P* < 0.05, ^**^ *P* < 0.01, ^***^ *P* < 0.001 vs. model group.

To evaluate the protective effect of GXNI on MI mice, the heart samples were weighed after 28 days of cardiac function tests, and H&E and Masson stainings were performed. As shown in Figure 2A, compared to that of the sham, the model hearts were larger, the ventricular walls were significantly thinner, the heart weight was significantly increased, and the infarcted area was seen to be white due to ischemia. Compared with the model, the GXNI- and MP-treated hearts were significantly smaller, weighed less, and had reduced white infarcted areas (Figures 2A–D). Masson staining showed that myocardial fibers in the sham aligned smoothly, and there were no obvious blue collagen fibers in the cell interstices. In contrast, the cardiomyocytes in the model showed a larger size, fewer in number, a disturbed arrangement, and significantly increased collagen deposition area. Drug administration decreased the collagen deposition, with the most significant effect observed in the GXNI high-dose and MP (Figures 2C,E). Immunofluorescence results in Figures 2F,G showed that only a small amount of α-SMA, a marker of myofibroblast activation, was expressed in the sham. MI significantly increased the expression of α-SMA in the model, particularly in the infarct area, whereas it was significantly reduced after treatment with GXNI and MP, indicating that GXNI can inhibit myofibroblast activation (Figure 2G). As shown in Figures 2H–J, the expression of myocardial fibrosis-related markers *Col1a1*, *Col3a1*, and *α-SMA* was significantly increased in the model compared with the sham. At the same time, GXNI and MP significantly decreased their expression.

### 3.3 Analysis of key targets of GXNI inhibition of myocardial fibrosis after MI

**FIGURE 3:**
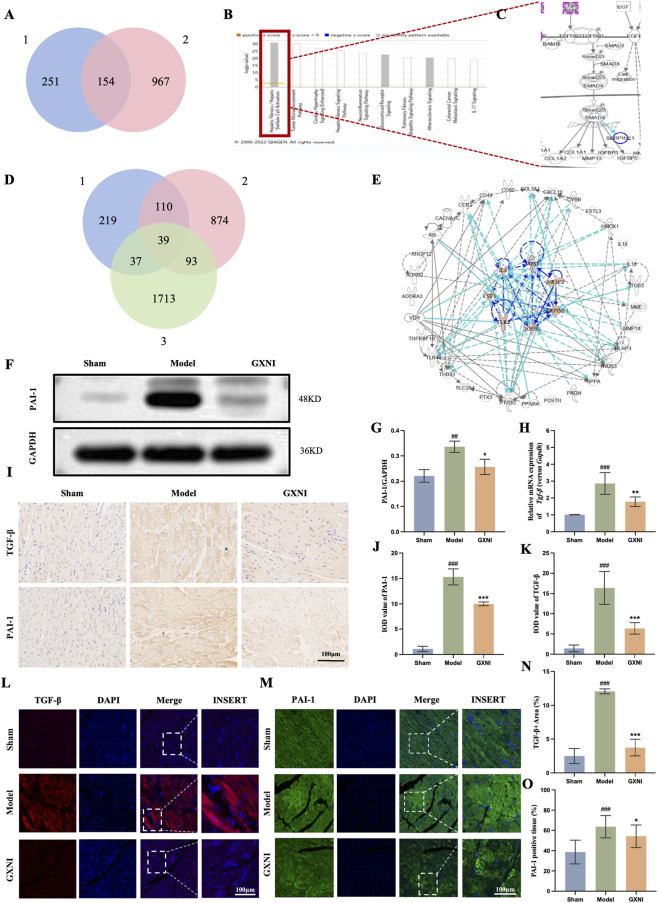
TGF-β1/PAI-1 is a key target of GXNI to inhibit myocardial fibrosis after MI. **(A)** Venn diagram of the “myocardial infarction” and “myocardial fibrosis” target libraries. **(B)** Top 10 typical pathways of disease through IPA core analysis. **(C)** Fibrosis-related pathways. **(D)** Venn diagram of “myocardial infarction” target library, “myocardial fibrosis” target library, and “cardiac GXNI vs. MI” transcriptome dataset. **(E)** Protein-protein interaction network diagram. **(F)** Representative WB image of PAI-1 and GAPGH for myocardial tissue in sham, model, and 3 mL/kg GXNI groups. **(G)** Quantification of PAI-1 WB results (n = 3). **(H)** The *Tgf-β1* mRNA expression levels (n = 3). **(I)** Representative images of IHC staining of PAI-1 and TGF-β1 for mouse myocardial tissue in sham, model, and 3 mL/kg GXNI groups. **(J–K)** Quantification of PAI-1 and TGF-β1 IHC results (n = 3). **(L)** Immunofluorescence staining of TGF-β1 in the mouse heart. **(M)** Immunofluorescence staining of PAI-1 in the mouse heart. **(N)** Quantification of TGF-β1 positive area in the mouse heart (n = 3). **(O)** Quantification of TGF-β1 positive area in the mouse heart (n = 3). Data were expressed as mean ± SD. Data were expressed as mean ± SD. ^##^ *P* < 0.01, ^###^ *P* < 0.001 vs. sham, ^*^ *P* < 0.05, ^**^ *P* < 0.01, ^***^ *P* < 0.001 vs. model. Scale bar = 100 µm.

Network pharmacology analysis identified 1,121 targets for “myocardial infarction” and 405 targets for “myocardial fibrosis”. Their intersection resulted in 154 common targets (Figure 3A). The top-ten pathways of these common targets by IPA Core analysis are listed according to the -log (P-value) score, including Hepatic Fibrosis/Hepatic Stellate Cell Activation, Tumor Microenvironment Pathway, Cardiac Hypertrophy Signaling, Hepatic Fibrosis Signaling Pathway, Neuroinflammatory Signaling Pathway, Glucocorticoid Receptor Signaling, Pulmonary Fibrosis Idiopathic Signaling Pathway, Atherosclerosis Signaling, Colorectal Cancer Metastasis Signaling, and IL-17 Signaling. The fibrosis-related pathways ranked high and were highly correlated with the fibrosis pathway (Figures 3B,C). The 1882 DEGs from our previous GXNI transcriptome datasets of MI mouse heart (Xiao et al., 2022) were intersected with the “myocardial infarction” and “myocardial fibrosis” targets, yielding 39 common targets (Figure 3D). IL-6, TGF-β1, SERPINE1 (PAI-1), NOS2, CSF3, and NR3C2 were identified as the core targets via the protein-protein interaction (PPI) network analysis (Figure 3E). Analysis of transcriptomic data, supported by existing literature, highlights SERPINE1 (PAI-1) as a compelling candidate within pro-fibrotic signaling networks and a target of significant therapeutic interest for myocardial fibrosis in recent years (Baumeier et al., 2021; Khan et al., 2021; Ricke-Hoch et al., 2020). TGF-β1 plays a key role in the process of myocardial fibrosis and acts as an upstream of PAI-1 in the fibrosis pathway, both of which constitute a pathway (Figures 3B,C), so the key target PAI-1 and its upstream TGF-β1 were chosen to be validated next. TGF-β1 and PAI-1 were found to be the key targets of GXNI to improve myocardial fibrosis after myocardial infarction by network pharmacology combined with transcriptomic data analysis.

To further verify the key role of the TGF-β1/PAI-1 pathway in the improvement of myocardial fibrosis after MI in mice by GXNI, protein expression levels of TGF-β1 and PAI-1 in cardiac tissue of MI mice were measured by WB, IHC, and PCR. Compared with the sham, the model mice exhibited a significant increase in the protein expression of both TGF-β1 and PAI-1. Consistent with this finding, the mRNA level of *Tgf-β1* was also markedly elevated. In contrast, GXNI significantly decreased the protein levels of TGF-β1 and PAI-1, and the mRNA level of *Tgf-β1* in the MI mouse heart (Figures 3F–K). Immunofluorescence results also showed that the positive areas of TGF-β1 and PAI-1 were significantly increased in the heart tissue of the MI model compared to the sham, and the positive expression was reduced after GXNI administration (Figures 3L–O), indicating that GXNI ameliorates fibrosis after myocardial infarction in mice by decreasing the expression of TGF-β1 and PAI-1.

### 3.4 Identification of PAI-1-interacting GXNI ingredients by molecular docking screen

**FIGURE 4:**
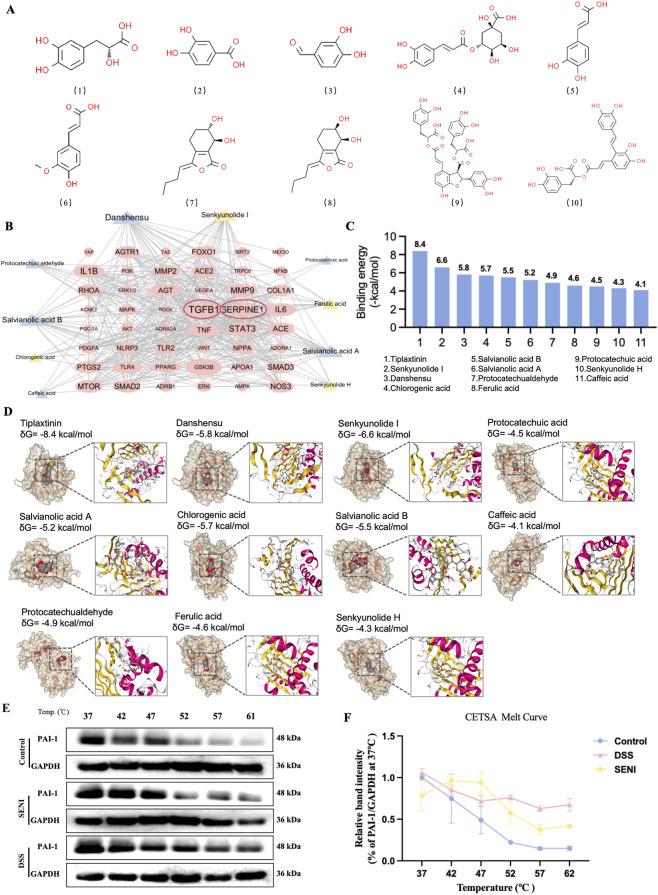
Ingredients in GXNI that bind to PAI-1. Structural-based binding prediction of GXNI ingredients to PAI-1 by molecular docking. **(A)** Chemical structures of the ten main active ingredients of GXNI. **(B)** Key GXNI active ingredient-disease target network. Nodes are color-coded to indicate their origin: Danshen ingredients are blue, Chuanxiong ingredients are yellow, and disease targets are pink. The size of each node is proportional to its degree value, reflecting the number of target interactions; a larger oval signifies that an active ingredient acts on a greater number of disease targets. TGF-B1 and SERPINE1 (PAI-1) are highlighted in red. **(C)** Relative affinity ranking of the ten main active ingredients of GXNI with PAI-1. **(D)** Molecular docking site map of the ten main active ingredients of GXNI and PAI-1. Tiplaxtinin, a PAI-1 inhibitor, served as a control. **(E)** Representative WB blots of cellular thermal shift assay. **(F)** The binding intensity curve of CETSA.

From our previous HPLC analysis and literature/database screen (Fan et al., 2025b; Ruan et al., 2012), we identified ten active GXNI components, danshensu, salvianolic acid B, salvianolic acid A, protocatechuic aldehyde, vanillin, tanshinone IIA, ferulic acid, rosmarinic acid, chlorogenic acid, and senkyunolide I, as candidates for PAI-1 partner (Figure 4A). IPA Protein-Protein Interaction (PPI) network analysis indicates that Danshensu from Danshen and Senkyunolide I from Chuanxiong both act on the key target PAI-1 and its upstream TGF-β1 (Figure 4B). In addition, the ten GXNI active ingredients were molecularly docked with PAI-1, useing Tiplaxtinin, a PAI-1 inhibitor, as a positive control. We found that these Senkyunolide I and Danshensu ranked in the top two regarding GXNI binding energy size (Figures 4C,D), suggesting that Senkyunolide I and Danshensu may serve as the key active components in GXNI that regulate TGF-β1/PAI-1 for the treatment of post-MI myocardial fibrosis. To further evaluate direct binding of Danshensu or Senkyunolide I to PAI-1, a cellular thermal shift assay (CETSA) was performed (Figure 4E), and the results demonstrated that treatment with either compound markedly increased the thermal stability of PAI-1 compared with the control (Figure 4F). Collectively, these findings indicate that Senkyunolide I and Danshensu stably interact with PAI-1, thereby validating the molecular docking prediction and supporting their role as principal mediators of GXNI inhibition of the TGF-β/PAI-1 fibrotic pathway.

### 3.5 GXNI and DSS + SENI reduced fibroblast activation and proliferation

**FIGURE 5:**
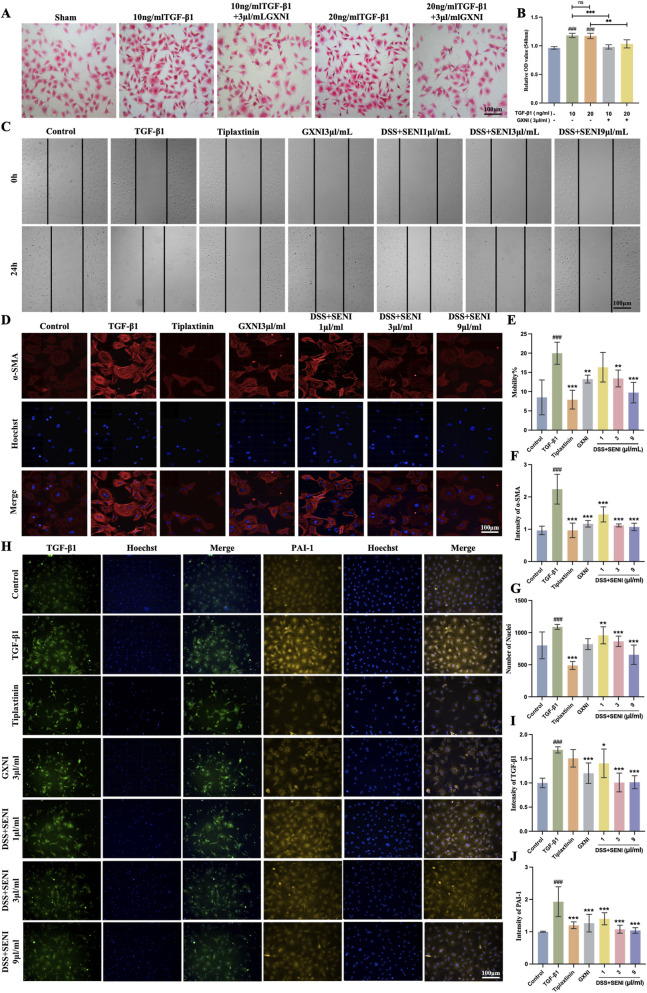
Effects of GXNI and DSS + SENI on TGF-β1-induced activation, migration, and proliferation of CFs. **(A)** Representative images of Sirius Red staining. **(B)** Quantification of collagen content (n = 3). **(C)** Changes in the migration of primary cardiac fibroblasts. **(D)** Representative images of fluorescence intensity of α-SMA. **(E)** 24 h fibroblast mobility (n = 3). **(F)** Quantification of α-SMA immunofluorescence staining. **(G)** Quantification of the number of primary cardiac fibroblast nuclei (n = 3). **(H)** Representative stained images of the nuclei (blue by Hoechst), the TGF-β1 protein (green), and the PAI-1 protein (yellow). **(I)** Quantification of the average fluorescence intensity of TGF-β1 (n = 3). **(J)** Quantification of the average fluorescence intensity of PAI-1 (n = 3). Data were expressed as mean ± SD. ^###^ *P* < 0.001 vs. control, ^**^ *P* < 0.01, ^***^ *P* < 0.001 vs. TGF-β1. Scale bar = 100 µm.

TGF-β1-induced cardiac fibroblast activation was examined using primary fibroblasts isolated from newborn rat hearts (a purity over 95% judged by Vimentin immunofluorescence staining, Supplementary Figure S2) and Sirius Red Collagen staining. The results in Figure 5A showed 3 μL/mL GXNI achieved the same inhibitory effect of fibroblast activation induced by either 10 ng/mL or 20 ng/mL TGF-β1, so the subsequent experiments were conducted using the modeling TGF-β1concentration as 10 ng/mL. Compared with the control, 10 ng/mL TGF-β1 effectively induced cardiac fibroblast migration after 24 h (Figure 5B). Similar to that of the PAI-1 inhibitor Tiplaxtinin, both GXNI and DSS + SENI inhibited the migration of cardiac fibroblasts, with the later exhibited a dose dependency (Figures 5C,D). Increased α-SMA expression and the number of nuclei in the TGF-β1 model, as revealed by immunofluorescence staining, indicated fibroblast activation and proliferation (Figure 5E). Tiplaxtinin, GXNI, and DSS + SENI all attenuated the mean fluorescence intensity of α-SMA and inhibited the proliferation of fibroblasts after administration, with the DSS + SENI combination showing a dose-dependent effect (Figures 5E–G). *In vitro* verification of the mechanism of action of GXNI and DSS + SENI (Figure 5H) showed that the expression of TGF-β1 and PAI-1 increased significantly in the model, while Tiplaxtinin weakly inhibited upstream TGF-β1 expression and significantly inhibited PAI-1 expression. GXNI and DSS + SENI both reduced myocardial fibrosis by inhibiting the expression of TGF-β1 and PAI-1 in cardiac fibroblasts *in vitro*, with the DSS + SENI combination showing a dose-dependent effect (Figures 5H–J).

### 3.6 The effects of DSS + SENI on TGF-β1/PAI-1 pathway gene expression and myocardial fibrosis in cryoinjured cardiac organoids

**FIGURE 6:**
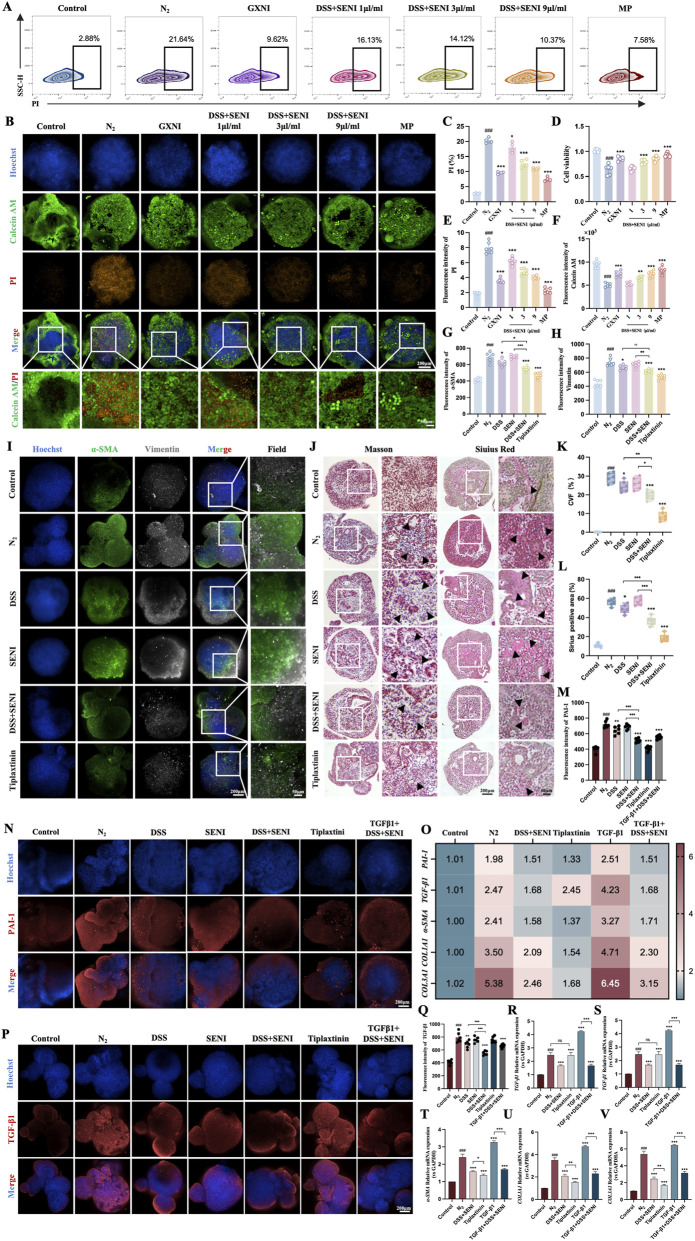
DSS SENI protects against myocardial fibrosis by regulating the TGF-β1/PAI-1 pathway in cryoinjury organoid. **(A)** Representative images of PI flow cytometry. **(B)** Representative images of fluorescent staining of cardiac organoids for Calcein AM and PI in each group. **(C)** PI-positive cells for heart organoids by flow cytometry analysis **(D)** Quantification of the cell viability, **(E)** average fluorescence intensity of calcein AM, and **(F)** average fluorescence intensity of PI (n = 6). **(G,H)** Quantification of the fluorescence intensity of α-SMA and PAI-1 (n = 6). **(I)** Representative images of immunofluorescence staining of α-SMA and PAI-1 in cardiac organoids. Representative chart of Sirius Red staining. **(J)** Representative images of Masson and Sirius Red staining of organoids. **(K,L)** Quantification of the collagen deposition area and Sirius positive area (n = 6). **(M)** Quantification of the average fluorescence intensity of PAI-1 in each group (n = 6). **(N)** Representative images of immunofluorescence staining of PAI-1 in organoids. **(O)** Representative gene expression by qPCR. **(P)** Representative images of immunofluorescence staining of TGF-β1 in organoids. **(Q)** Quantification of the average fluorescence intensity of TGF-β1 in each group (n = 6). **(R–V)** Quantification of *PAI-1*, *TGF-β1*, *α-SMA*, *COL1A1,* and *COL3A1* mRNA expression in each group (n = 3). Data were expressed as mean ± SD. ^###^ *P* < 0.001 vs. control, ^**^ *P* < 0.01, ^***^ *P* < 0.001 vs. N_2_ model. Scale bar = 200 µm.

Flow cytometry was used to assess the cardiac injury and apoptosis in N_2_ cryoinjured organoids. As shown in Figure 6A, the mean fluorescence intensity of PI in the model significantly increased compared to the control (Figure 6C). GXNI, DSS + SENI (1, 3, and 9 μL/mL) and MP significantly reduced the apoptosis in the heart organoid (Figure 6C). Calcein AM and PI fluorescent staining indicated that N_2_ cryoinjury decreased cell viability and membrane integrity, whereas GXNI, DSS + SENI (1, 3, and 9 μL/mL), and MP all increased cell viability and membrane integrity (Figures 6B,D,E,F). Since a concentration of 3 μL/mL DSS + SENI effectively protected cardiac organoids from cryoinjury damage, it was chosen as the dose for subsequent experiments.

To address the question if the protective effect of DSS + SENI in the cryoinjury model was due to a combinatorial effect of DSS and SENI, functional analysis through heart-specific proteins and pathological evaluation were performed (Figures 6I,J). Fluorescence intensity of α-SMA and Vimentin increased significantly after cryoinjury, while the DSS + SENI and Tiplaxtinin significantly inhibited α-SMA and Vimentin expression (Figure 6I). It is worth noting that although DSS or SENI alone somewhat reduced the elevated α-SMA and Vimentin levels, it is their combination that markedly suppresses the fibrotic marker protein expression (Figures 6G,H). Pathological evaluation by Masson and Sirius Red stains of the anti-myocardial fibrosis effect of DSS + SENI (Figure 6J) showed that DSS + SENI markedly decreased cardiac fibrosis and collagen in cryoinjury organoids (Figures 6K,L), while Tiplaxtinin, as a PAI-1 inhibitor, also significantly reduced the fibrotic area compared with the cryoinjury model. Specifically, DSS alone significantly ameliorated myocardial fibrosis and collagen deposition, whereas SENI alone showed a trend but not a statistically significant reduction. Notably, similar to Tiplaxtinin, the combination of DSS and SENI achieved superior effects compared with either agent alone (Figures 6K,L). These results suggest that DSS and SENI exert combinatorial effects in mitigating pathological changes.

To further demonstrate that DSS + SENI inhibits cardiac fibrosis through the TGF-β1/PAI-1 pathway, we examined the combination effect of TGF-β1 and DSS + SENI. Immunofluorescence showed that cryoinjury reduced the expression of TGF-β1 and PAI-1 (Figures 6N,P). As shown above, compared with the N_2_ cryoinjury model, treatment with DSS + SENI and combined TGF-β1 and DSS + SENI decreased the expression of TGF-β1 and PAI-1, respectively. Specifically, DSS alone significantly suppressed the elevated expression of TGF-β1 and PAI-1, whereas SENI alone exhibited a trend toward but not a statistically significant reduction. The combination of DSS and SENI achieved superior effects compared with either agent alone (Figures 6M,N,P,Q). It is noteworthy that the PAI-1 inhibitor Tiplaxtinin also significantly inhibited the expression of PAI-1, but not that of TGF-β1, indicating that TGF-β1 is upstream of PAI-1 (Figures 6P,Q). Interestingly, the combination of TGF-β1 and DSS SENI significantly inhibited the expression of TGF-β1 and PAI-1, suggesting that DSS SENI protects hypothermic-injured cardiac organoids through the TGF-β1/PAI-1 pathway (Figures 6P,Q). The results of QPCR analysis (Figure 6O) are similar to those of immunofluorescence. Compared with the control, N_2_ cryoinjury increased the expression of *TGF-β1*, *PAI-1*, *α-SMA*, *COL1A1*, and *COL3A1* mRNA. DSS + SENI and combined TGF-β1 and DSS + SENI significantly reduced the mRNA overexpression of *TGF-β1*, *PAI-1*, *α-SMA*, *COL1A1*, and *COL3A1*. Tiplaxtinin decreased the overexpression of *PAI-1*, *α-SMA*, *COL1A1*, and *COL3A1*, but not significantly of *TGF-β1,* in cryoinjury (Figures 6R–V). Importantly, the recombinant TGF*-β1* treatment significantly increased the mRNA expression of *TGF-β1*, *PAI-1*, *α-SMA*, *COL1A1*, and *COL3A1*, whereas the combined TGF-β1 and DSS + SENI treatment inhibited their overexpression. Hence, DSS + SENI improved myocardial fibrosis after cryoinjury by inhibiting the expression of the TGF-β1/PAI-1 pathway in cardiac organoids *in vitro*.

## 4 Discussion

Combining animal, cell, and three-dimensional organoid models, this study demonstrates that GXNI ameliorates myocardial fibrosis post-myocardial infarction by suppressing the TGF-β1/PAI-1 pathway. Firstly, transcriptomic and IPA analyses in a murine permanent LAD ligation model identified this mechanism. Molecular docking and direct binding experiments validated Danshensu and Senkyunolide I as the primary active components of GXNI. Consistently, both GXNI and the compound combination suppressed TGF-β1-induced fibroblast activation, proliferation, and migration *in vitro*. Finally, studies in the cryoinjury model to IPSC-induced organoids, which better simulate myocardial infarction, demonstrated that DSS + SENI improves post-infarction myocardial fibrosis by combinatorially regulating the TGF-β1/PAI-1 pathway.

Myocardial cell injury and myocardial fibrosis induced after myocardial infarction are the two main mechanisms in the process of cardiac remodeling and the main pathological changes in the development of heart failure after myocardial infarction (Bacmeister et al., 2019). Data from a large number of clinical studies indicate that after myocardial infarction, timely cardiac repair and inhibition of myocardial fibrosis are important strategies to prevent further myocardial damage and deterioration of cardiac function (Gyongyosi et al., 2017). When the heart suffers an injury, cardiac fibroblasts are activated and subsequently differentiate into myofibroblasts, which are the main mediators in the cardiac pathological remodeling process. Activated myofibroblasts have proliferative and migratory properties, leading to extracellular matrix expansion as well as excessive deposition of collagen. It then leads to fibrotic scarring and cardiac insufficiency, and even induces heart failure when myofibroblasts continue to act (Liu et al., 2021; Venugopal et al., 2022). Currently, the treatment of myocardial fibrosis after myocardial infarction remains an unresolved problem. In this study, we demonstrated that GXNI reduced cardiac injury, decreased the area of myocardial infarction and collagen deposition, and inhibited fibroblast activation to improve myocardial fibrosis after MI in mice and cryoinjury heart organoids. This finding is an extension of the earlier works by us and others, that GXNI ameliorates cardiac remodeling in HF mouse and 3 days heart spheroid models via p38/fos/mmp1-mediated inhibition of myocardial hypertrophy and fibrosis (Fan et al., 2023). Wang C et al. showed that GXNI alleviates fibrosis in heart failure mice and regulates the SLC7A11/GPX4 axis (Wang et al., 2023). It is important to emphasize that while post-MI fibrosis and HF-associated fibrosis represent a pathological continuum, they are fundamentally distinct in their initiation, developmental patterns, and functional consequences (Zhang et al., 2024)—a nuance that underscores the potential value of GXNI in intervening at multiple stages of fibrotic heart disease. In the MI context examined in this study, fibrosis is triggered primarily by immune cell and cardiomyocyte necrosis, manifesting as an inflammatory response, phagocytosis, reparative, and replacement process aimed at forming a stable scar to maintain structural integrity (Gissler et al., 2024). Its evolution follows a defined sequence of inflammatory, proliferative, and maturation phases. In contrast, in the chronic HF setting investigated in our earlier research, fibrosis transitions into a diffuse, reactive, and pathological process. This form is driven by persistent neurohormonal activation and hemodynamic stress by interstitial and perivascular collagen accumulation that increases myocardial stiffness and impairs diastolic function (Noels et al., 2025). Thus, while post-MI fibrosis is characterized by regional scar formation that initially preserves structural continuity but impairs regional systolic function and initiates remodeling, HF-related fibrosis is marked by global interstitial infiltration, which predominantly compromises diastolic compliance, promotes arrhythmogenicity, and accelerates disease progression toward advanced HF. The efficacy of GXNI in attenuating fibrosis in both pathological contexts—acute infarction and chronic failure—suggests its ability to modulate common downstream pathways critical to myofibroblast activation and collagen turnover. This positions GXNI as a promising broad-spectrum anti-fibrotic candidate and provides experimental evidence supporting its role in mitigating the dynamic evolution of fibrosis from initial injury to chronic heart failure.

The level of fibrinogen activator inhibitor type 1 (PAI-1) is an important indicator in clinical testing of patients with acute myocardial infarction (Abd El-Aziz and Rezk, 2015; Collet et al., 2003; Soeki et al., 2000), and it is one of the main substances that leads to the reduction of fibrinolytic activity in the circulation and thus to thrombosis. It has an important impact on stroke, heart attack, and atherosclerosis (Van De Craen et al., 2012; Vaughan, 2005). Usually, serum expression levels of PAI-1 are elevated in patients with acute myocardial infarction, and the higher the level of PAI-1 in patients, the greater the degree of coronary artery disease (Shimizu et al., 2016). Meanwhile, PAI-1, a downstream target of TGF-β1, is closely associated with fibrosis in a variety of organs (Flevaris and Vaughan, 2017; Ghosh and Vaughan, 2012; Rabieian et al., 2018). Excessive deposition of extracellular matrix due to increased collagen synthesis and reduced collagen degradation by protein hydrolase activity is one of the characteristics of fibrosis. In contrast, the PAI-1-regulated tissue-type/uremic kinase-type fibrinogen activator (tPA/uPA) is an important factor in regulating protein hydrolase activity. High expression of PAI-1 leads to the formation of collagen and other ECM proteins at the wound scarring, and inhibition of PAI-1 expression reduces organ fibrosis (Ghosh and Vaughan, 2012). It has been suggested that PAI-1 may be an important target of lung cell senescence-induced pulmonary fibrosis (Adnot et al., 2020; Huang et al., 2012; Rana et al., 2020). In addition, TGF-β1 induces PAI-1 expression in esophageal epithelial cells, thus leading to esophageal fibrosis (Rawson et al., 2016). Thrombin increases PAI-1/MMT expression in pleural mesothelial cells by regulating PAR-1 signaling, leading to pleural fibrosis in tuberculous pleural effusions (Hsieh et al., 2019). However, PAI-1’s role in myocardial fibrosis remains controversial, with some studies showing that it binds to bilirubin, attenuating myocardial fibrosis (Zhong et al., 2014), and PAI-1 knockdown leads to cardiac fibrosis in aged mice, which may be related to the lack of PAI-1 production and secretion in cardiomyocytes (Ghosh et al., 2023). Nevertheless, it has also been shown that overexpression of PAI-1 leads to myocardial fibrosis after myocardial infarction (Li et al., 2016; Takeshita et al., 2004), and that PAI-1 has a pro-fibrotic effect in the infarcted heart (Zaman et al., 2009). Loureirin B can also attenuate myocardial fibrosis and thus improve myocardial ischemia/reperfusion injury by inhibiting the PAI-1/TGF-β1/Smad signaling pathway (Kong and Zhao, 2022). Therefore, the mechanism of action of PAI-1 in myocardial fibrosis still leaves much to be desired. Our network pharmacological analysis, combined with reassessing the transcriptomic data showed that the effect of GXNI on improving myocardial fibrosis after myocardial infarction may be achieved by regulating TGF-β1/PAI-1. We also found that Danshensu from Danshen and Senkyunolide I from Chuanxiong may be the key active GXNI components in the regulation of TGF-β1 and PAI-1, which were verified in primary fibroblasts and heart organoids. The mechanism by which PAI-1 influences myocardial fibrosis, however, remains incompletely resolved, as evidenced by the seemingly contradictory findings in the literature. This apparent duality may be best understood within the context-dependent “double-edged sword” paradigm of PAI-1 biology. In the acute phase of MI, a transient increase in PAI-1 may initially play a protective role by temporally stabilizing the provisional matrix and modulating cell migration (Ghosh et al., 2023). However, in the subsequent chronic remodeling phase, persistently elevated PAI-1, often driven by sustained TGF-β1 signaling, shifts decisively towards a pro-fibrotic role (Vaughan, 2006). This is primarily mediated by its inhibition of the plasmin/MMP proteolytic cascade, effectively suppressing ECM degradation and promoting net collagen accumulation. Our finding that GXNI downregulates both TGF-β1 and PAI-1 suggests its action may lie in interrupting this dominant chronic pro-fibrotic axis (Moideen et al., 2024). The identification of Danshensu and Senkyunolide I as putative active components further supports a multi-target strategy to rebalance the dysregulated protease landscape, favoring resolution over progression of fibrosis.

It should be noted that the 28-day post-MI endpoint selected in this study unequivocally corresponds to the chronic remodeling phase in the mouse model. Previous work has established that the first 3–7 days after MI represent the phase of acute inflammation and early granulation tissue formation, whereas from day 14 onward, the heart enters a chronic remodeling stage characterized by scar maturation and interstitial fibrosis (Prabhu and Frangogiannis, 2016). Therefore, at this 28-day chronic time window, the elevated expression of TGF-β1 and PAI-1, together with the pathological manifestations of myocardial fibrosis, reflects persistent, chronic pro-fibrotic signaling rather than an acute-phase compensatory response. This temporal positioning provides a critical chronological framework for our subsequent discussion that, in the chronic stage, PAI-1 predominantly assumes a pro-fibrotic role. Accumulating evidence indicates that the role of PAI-1 in myocardial fibrosis is highly time- and context-dependent, embodying a prototypical “double-edged sword” paradigm. In the acute post-MI phase, a moderate increase in PAI-1 is protective. PAI-1-deficient mice display heightened vascular permeability, excessive inflammation, and spontaneous cardiac fibrosis, underscoring that basal PAI-1 is indispensable for microvascular integrity and cardiac homeostasis (Xu et al., 2010). In the chronic remodeling phase, however, sustained PAI-1 upregulation exerts profibrotic effects principally by inhibiting the plasmin/MMP proteolytic cascade, thereby promoting excessive collagen and ECM accumulation (Ghosh and Vaughan, 2012). Flevaris and Vaughan further highlight an organ-specific paradox: while PAI-1 deficiency attenuates fibrosis in the liver, lung, and kidney, it paradoxically promotes age-dependent spontaneous cardiac fibrosis (Flevaris and Vaughan, 2017). A mechanistic explanation emerges from cardiomyocyte-specific PAI-1 knockout mice, in which loss of PAI-1-mediated suppression of TGF-β synthesis leads to TGF-β pathway overactivation, profound transcriptomic alterations, and exaggerated fibrosis under pressure overload (Ghosh et al., 2023). Thus, PAI-1 serves as an intracellular negative regulator of TGF-β production in cardiomyocytes, while its extracellular anti-proteolytic activity drives matrix accumulation. This dual functionality explains why PAI-1 appears antifibrotic in some settings yet profibrotic in others. Collectively, PAI-1 acts as a temporal double-edged sword, protective during the acute phase through provisional matrix stabilization, but pathogenic in the chronic phase by perpetuating ECM accumulation. As our study endpoint (28 days post-MI) unequivocally falls within the chronic profibrotic stage, the observed downregulation of the TGF-β1/PAI-1 axis by GXNI likely reflects therapeutic intervention in this dominant profibrotic signaling, thereby attenuating myocardial fibrosis. Although longitudinal comparisons across different post-MI time points have not yet been conducted, future studies should be designed to systematically address stage-specific regulation of PAI-1 by GXNI, Danshensu, and Senkyunolide I. Based on current evidence, Danshensu antagonizes cardiac fibrosis through ROS/p38 MAPK inhibition (Lu et al., 2014), while Senkyunolide I exhibits anti-inflammatory, antioxidative, and emerging antifibrotic activities (Huang et al., 2023). Since ROS and inflammatory signaling are key drivers of PAI-1 expression across both acute and chronic phases, we speculate that these compounds may differentially modulate PAI-1 by attenuating such upstream stressors: predominantly restraining TGF-β1-driven persistent PAI-1 in the chronic phase to limit fibrosis, while mildly regulating PAI-1 in the acute phase to preserve its vasoprotective functions.

TGF-β1 is an important mediator in a variety of fibrotic processes, mediating fibroblast to myofibroblast conversion and the deposition of collagen (Lan, 2011; Peng et al., 2022). We established a stable *in vitro* method for primary cardiac fibroblast extraction and induced myocardial fibrosis model by stimulating primary cardiac fibroblasts with TGF-β1. Both GXNI and monomeric combinations of Danshensu and Senkyunolide I were shown to inhibit the activation, proliferation, and migration of primary cardiac fibroblasts and inhibit fibrosis by scratch assays and immunofluorescence assays. Meanwhile, the combination of GXNI with Danshensu and Senkyunolide I has also been shown to reduce cell necrosis in heart organoids, and the combination of Danshensu and Senkyunolide I monomers with recombinant TGF-β1 significantly inhibits the overexpression of TGF-β1 and PAI-1, indicating that DSS + SENI protects against post-myocardial infarction myocardial fibrosis by regulating the TGF-β1/PAI-1 pathway. To substantiate our mechanistic findings, we employed a novel *in vitro* cryoinjury model in heart organoids to simulate post-MI remodeling, moving beyond conventional two-dimensional TGF-β1 stimulation. While the oxygen/glucose deprivation and reperfusion (OGD/R) model fittingly recapitulates the metabolic and apoptotic cascades of acute ischemia, the cryoinjury model offers a distinct and complementary advantage for fibrosis research (Mamic et al., 2021; Voetmann et al., 2022). It induces focal, physical necrosis, thereby more faithfully mimicking the spatial progression and wound healing timeline observed in vivo—from initial cell death and inflammatory recruitment to robust granulation tissue formation and collagen-based scar maturation (Tiburcy et al., 2017). This model is increasingly recognized for its high fidelity in modeling the post-infarction fibrotic phase that directly leads to adverse ventricular remodeling and heart failure. Recent studies employing similar 3D engineered heart tissues have demonstrated that cryoinjury effectively triggers a sustained pro-fibrotic signaling cascade, including marked TGF-β1 activation, making it a superior platform for evaluating anti-fibrotic interventions like GXNI (Hofbauer et al., 2021; Ikushima et al., 2022). Our observation that GXNI ameliorates fibrosis in this model strengthens the translational relevance of our findings, as it demonstrates efficacy in a system that closely replicates the integrated biological processes of scar formation following MI.

While this study provides a comprehensive evaluation of GXNI across murine, primary cell, and organoid models, several limitations should be acknowledged. First, although the key component combination (Danshensu and Senkyunolide I) was identified, their potential combinatorial interactions were only qualitatively, but not quantitatively, elucidated. Future studies of high-throughput combinatorial screening are needed to explore the pharmacological synergistic effects and specific synergistic mechanisms of GXNI and to reveal their pairing significance. Second, the employed cardiac organoid model, while superior to 2D cultures, lacks an integrated immune component, thereby failing to fully recapitulate the complex post-MI inflammatory and fibrotic microenvironment. To address this, the development of more sophisticated multicellular organoids incorporating immune cells, such as macrophages, would be a valuable direction for future research, offering a more physiologically relevant platform for drug discovery and reducing the reliance on animal models. Third, while the discussion acknowledges the context-dependent protective versus pro-fibrotic roles of PAI-1, the present study did not examine the temporal dynamics of PAI-1 expression following myocardial infarction. Time-course data are essential to distinguish whether GXNI modulates the early protective phase versus the chronic pro-fibrotic axis of PAI-1. The lack of such temporal resolution limits our ability to definitively attribute the anti-fibrotic effect of GXNI specifically to suppression of the maladaptive, pro-fibrotic PAI-1 pathway. Future studies incorporating sequential sampling post-MI are warranted to address this gap and further strengthen the mechanistic interpretation of GXNI’s action on PAI-1.

## 5 Conclusion

**FIGURE 7:**
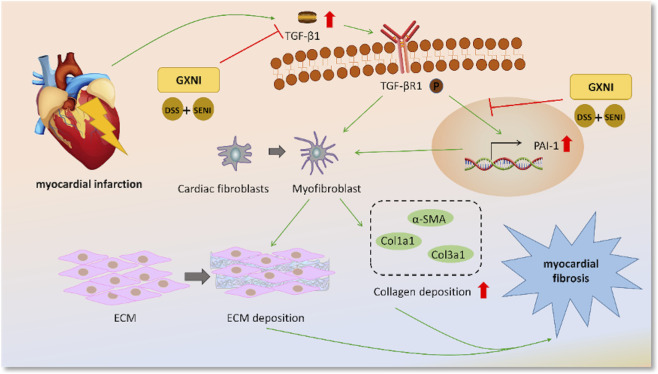
Schematic diagram of the mechanism by which GXNI modulates TGF-β1/PAI-1 to improve myocardial fibrosis after myocardial infarction in mice.

Our study demonstrates that GXNI may improve myocardial fibrosis after myocardial infarction, at least in part by modulating TGF-β1/PAI-1 (Figure 7), and that the main active components of its action are Danshensu and Senkyunolide I. The study provides new evidence for the role of PAI-1 in myocardial fibrosis and a theoretical basis for the clinical use of GXNI in the treatment of myocardial fibrosis after myocardial infarction.

## Data Availability

The original contributions presented in the study are included in the article/Supplementary Material, further inquiries can be directed to the corresponding author/s.
