# Supplementary material for: Senkyunolide I and Danshensu in combination ameliorate post-MI myocardial fibrosis by regulating TGF-β1/PAI-1 in mice and heart organoids
Source: Front Pharmacol. 2026 Jul 8;17:1862471. doi: 10.3389/fphar.2026.1862471 (PMC13388070; doi:10.3389/fphar.2026.1862471)
Supplement: Supplementary file 1 [file Supplementaryfile1.docx]

**Supplemental Information**


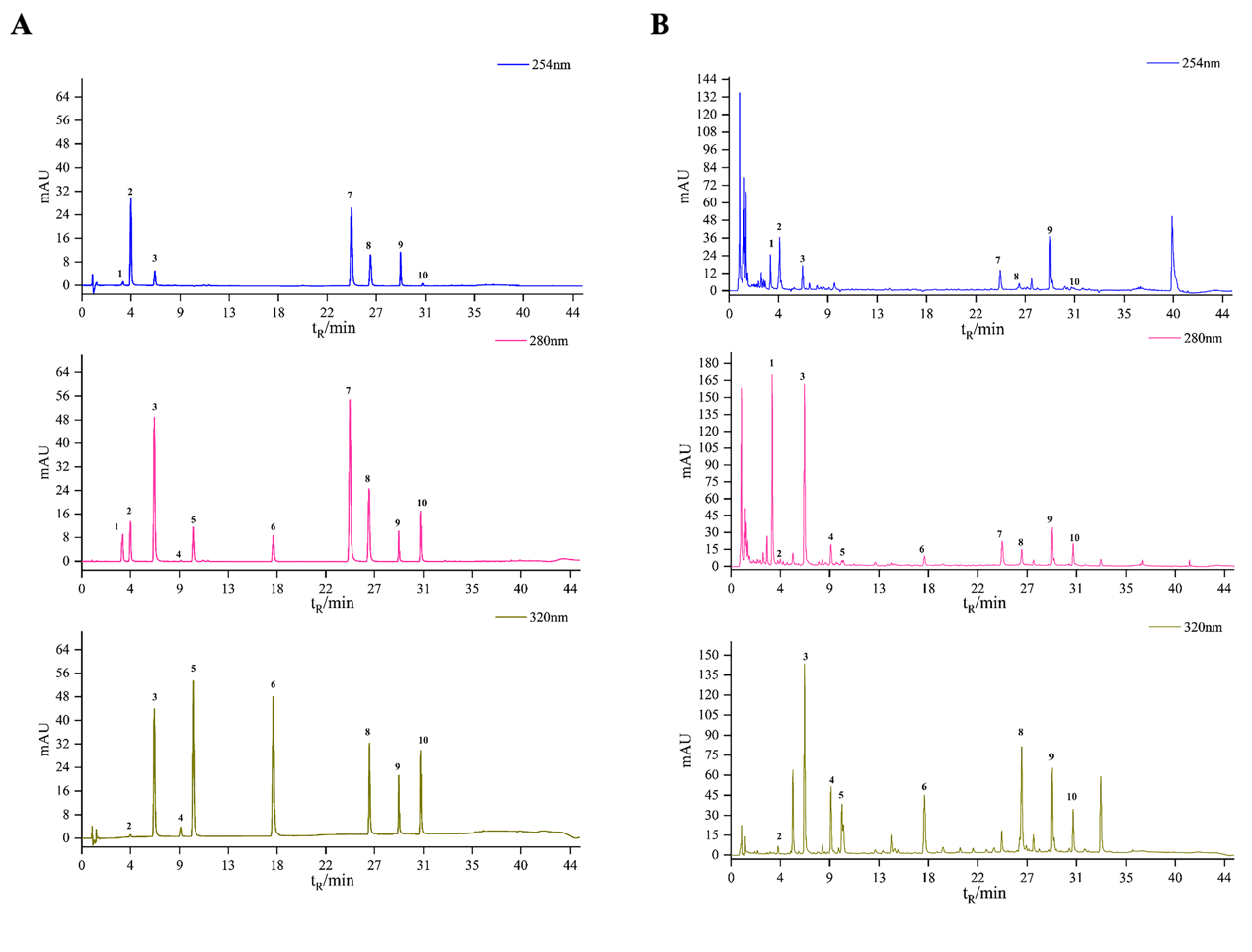


**Figure S1. The HPLC fingerprint chromatogram of GXNI (A) and (B) represent the chromatograms of the mixed reference substance and sample, while 1, 2, and 3 represent chromatographic information collected at wavelengths of 254nm, 280nm and 320nm, respectively.**

Peak number: 1. Danshensu; 2. Protocatechuic acid; 3. Protocatechualdehyde; 4. Chlorogenic acid; 5. Caffeic acid; 6. Ferulic acid; 7. Senkyunolide I; 8. Senkyunolide H; 9. Salvianolic acid B; 10. Salvianolic acid A.

**
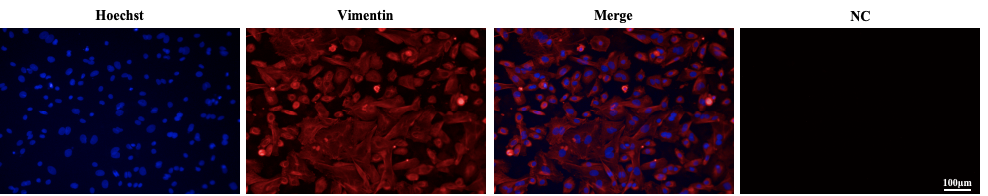
**

**Figure S2 Representative images of immunofluorescence staining of Vimentin (fibroblast).** Scale bar = 100 µm.
